# Phosphodiesterase 3B Is Localized in Caveolae and Smooth ER in Mouse Hepatocytes and Is Important in the Regulation of Glucose and Lipid Metabolism

**DOI:** 10.1371/journal.pone.0004671

**Published:** 2009-03-05

**Authors:** Karin Berger, Rebecka Lindh, Nils Wierup, Emilia Zmuda-Trzebiatowska, Andreas Lindqvist, Vincent C. Manganiello, Eva Degerman

**Affiliations:** 1 Department of Experimental Medical Sciences, Lund University, BMC C11, Lund, Sweden; 2 Department of Experimental Medical Sciences, Lund University, BMC B11, Lund, Sweden; 3 Translational Medicine Branch, National Heart, Lung, and Blood Institute (NHLBI), National Institutes of Health (NIH), Bethesda, Maryland, United States of America; Sapienza University of Rome, Italy

## Abstract

Cyclic nucleotide phosphodiesterases (PDEs) are important regulators of signal transduction processes mediated by cAMP and cGMP. One PDE family member, PDE3B, plays an important role in the regulation of a variety of metabolic processes such as lipolysis and insulin secretion. In this study, the cellular localization and the role of PDE3B in the regulation of triglyceride, cholesterol and glucose metabolism in hepatocytes were investigated. PDE3B was identified in caveolae, specific regions in the plasma membrane, and smooth endoplasmic reticulum. In caveolin-1 knock out mice, which lack caveolae, the amount of PDE3B protein and activity were reduced indicating a role of caveolin-1/caveolae in the stabilization of enzyme protein. Hepatocytes from PDE3B knock out mice displayed increased glucose, triglyceride and cholesterol levels, which was associated with increased expression of gluconeogenic and lipogenic genes/enzymes including, phosphoenolpyruvate carboxykinase, peroxisome proliferator-activated receptor γ, sterol regulatory element-binding protein 1c and hydroxyl-3-methylglutaryl coenzyme A reductase. In conclusion, hepatocyte PDE3B is localized in caveolae and smooth endoplasmic reticulum and plays important roles in the regulation of glucose, triglyceride and cholesterol metabolism. Dysregulation of PDE3B could have a role in the development of fatty liver, a condition highly relevant in the context of type 2 diabetes.

## Introduction

Cyclic nucleotide phosphodiesterases (PDEs) are important regulators of signal transduction processes mediated by cAMP and cGMP. The PDE family contains eleven structurally related and functionally distinct subfamilies (PDE1-11) that differ in their primary structures, affinities for cAMP and cGMP, responses to specific effectors and inhibitors, as well as mechanisms through which they are regulated [1]. PDE3 isoforms are encoded by two similarly organized genes, PDE3A and PDE3B. These enzymes hydrolyze cAMP and cGMP with high affinity in a mutually competitive manner and are inhibited by compounds such as cilostamide, cilastazol and milrinone [2], [3]. The structural organization of PDE3A and PDE3B proteins is identical with the catalytic domain found in all PDEs located in the C-terminal portions of the molecules [2], [3]. The catalytic domains of PDE3A and B are highly conserved, except for an insertion of 44 unique amino acids that is not found in the catalytic domains of other PDE families and that also differs in, and thus distinguishes, PDE3A and B isoforms [2], [3]. Their N-terminal regulatory domains are quite divergent, consisting of two hydrophobic regions important for membrane association of these enzymes. Full-length PDE3s (Mw 135 kDA) are found in association with membranes; smaller PDE3A forms are found in cytosolic fractions [4]. Furthermore, PDE3B has been shown to be localized to the endoplasmic reticulum (ER) and to specific detergent-resistent parts of the plasma membrane, caveolae [5], [6]. Caveolae are special forms of lipid rafts observed as small flask-shaped 50–100 nM invaginations of the plasma membranes and are particularly abundant in adipocytes. They have a high content of sphingolipids, cholesterol and are stabilized by one or more isoforms of caveolin. Caveolae are believed to be important in the organization of signal transduction events, particularly insulin and cAMP signalling [7]. The exact intracellular location of the hepatocyte PDE3B has not been elucidated. The N-terminal region of PDE3B contains regulatory phosphorylation sites [2], [3]. Multisite phosphorylation of PDE3s has, for example, been demonstrated in adipocytes, hepatocytes and HeLa cells [8], [9] which is believed to be important in the regulation of PDE3 activity and in interaction with other proteins [2], [3].

PDE3A and PDE3B exhibit cell-specific differences in expression. PDE3A is highly expressed primarily in the cardiovascular system, for example in platelets, smooth muscle cells and cardiac myocytes [2], [3]. PDE3B on the other hand is relatively highly expressed in cells important in energy metabolism, such as white and brown adipocytes, pancreatic β-cells and liver [2], [3] indicating a role for this enzyme in the regulation of metabolism.

Recent results from PDE3B transgenic mouse models do indicate that PDE3B plays an important role in overall regulation of energy metabolism [10], [11]. For example, mice that specifically overexpress PDE3B in pancreatic β-cells demonstrate glucose intolerance and impaired insulin response to glucose and glucagon-like peptide-1 (GLP-1) [10]. The phenotype of PDE3B knock out (KO) mice is complex. Hence, on one hand PDE3B KO mice are lean and have improved insulin secretion but they also exhibit glucose intolerance, insulin resistance and increased lipolysis [11]. The role of hepatocyte PDE3B in the regulation of lipid and glucose metabolism remains unknown. However, clamp studies in PDE3B KO mice show increased glucose production and reduced ability of insulin to suppress glucose production indicating multiple roles for this enzyme in hepatocytes [11].

In this study we demonstrate that, in hepatocytes, PDE3B is localized to caveolae and smooth ER and that the enzyme has an important role in the regulation of triglyceride, cholesterol and glucose metabolism in these cells.

## Materials and Methods

### Ethics statement

All animals were handled in strict accordance with good animal practice as defined by the national and local animal welfare bodies, and all animal work was approved by the Ethics Committee at Lund University, Lund, Sweden.

### Materials

C57BL/6 male mice were purchased from Taconic (Skensved, Denmark). Caveolin-1 KO mice on the C57BL/6 background, obtained from the Jackson Laboratory (Bar Harbor, Maine, USA), were further backcrossed on the same background and genotyped as described by Razani et al [12]. PDE3B deficient mice were generated and characterized as previously described in [11] (a fragment containing exon 1 of the mouse *Pde3b* gene was cloned into the pBluescript and used to construct the targeting vector). Antibodies against caveolin-1 (rabbit polyclonal) and adenylyl cyclase (AC) V were obtained from Santa Cruz Biotechnology (Santa Cruz, CA, USA); flotillin-1, Na^+^K^+^-ATPase, nucleoporin p62 and BiP from BD Transduction Laboratories. Antibodies against phosphoenolpyruvate carboxylase [PEPCK (PCK1)] were obtained from Abgent (San Diego, CA, USA). Anti-PDE3B antibodies used for immunoblot analysis were raised in rabbits against the peptide CGYYGSGKMFRRPSLP and affinity purified. For electron microscopy affinity purified antibodies against the C-terminal (CT) part of PDE3B were generated in rabbit using the peptide NASLPQADEIQVIEEA. As secondary antibody, HRP-goat anti-rabbit antibody (BioSource, Invitrogen, Carlsbad, CA, USA) was used. If not otherwise stated, reagents and chemicals were obtained from Sigma-Aldrich (Stockholm, Sweden).

### Subcellular fractionation

Hepatocyte subcellular fractions were prepared according to Fleisher and Kervina [13] with a few modifications based on a study by Tuma et al [14]. Isolated and washed hepatocytes (35–100×10^6^ cells) from C57BL/6 mice or washed whole livers from 10–12 weeks old caveolin-1 KO and wild type (WT) mice were homogenized with a Dounce homogenizer (10–15 strokes) in 10 ml buffer A (10 mM HEPES pH 7.5, 0.25 M sucrose, 1 mM EDTA, 1 mM NaF, 0.2 mM sodium orthovanadate, 1 µg/ml pepstatin A, 10 µg/ml leupeptin and 10 µg/ml antipain). The homogenate was centrifuged (280×g, 5 min). The supernatant was saved and the pellet rehomogenized in 2.5 ml buffer A and centrifuged as above (280×g, 5 min). The first and second supernatants were combined and further centrifuged (1 500×g for 10 min). The resulting supernatant (sup 1) was further centrifuged as described below and the pellet was homogenized in 2 ml buffer B (10 mM Tris pH 7.4, 0.25 M sucrose, 0.5 mM MgCl_2_, 3 mM NaF, 0.6 mM sodium orthovanadate, 3 µg/ml pepstatin A, 30 µg/ml leupeptin and 30 µg/ml antipain). The homogenate was supplemented with 6.75 ml of a sucrose rich buffer (2 M sucrose, 10 mM Tris (pH 7–8) and 0.5 mM MgCl_2_ in order to yield a final concentration of 1.6 M sucrose. The suspension was transferred into a rotor tube, overlayed with 2 ml buffer B and centrifuged (SW41, 70 900×g, 70 min). The interphase with plasma membranes was collected and the pellet containing nuclei was rehomogenized in buffer A supplemented with sucrose to a final concentration of 2 M, and centrifuged (70 900×g, 60 min). The pellet, referred to as the nuclei fraction, was suspended in 1 ml buffer C (50 mM TES pH 7.4, 50 mM sucrose, 1 mM EDTA, 0.1 mM EGTA, 1 µg/ml pepstatin A, 10 µg/ml leupeptin and 10 µg/ml antipain). The interphase containing plasma membranes was resuspended in 6 ml buffer A and centrifuged (1 700×g, 10 min). The pellet was used for caveolae enrichment as described in the next section, or further purified with regard to the plasma membrane on a 1.45 M sucrose gradient according to Fleisher and Kervina [13]. The purified plasma membrane fraction was finally suspended in 1 ml buffer C. The supernatant from the first centrifugation step (sup1) was centrifuged twice (8 000×g, 15 min) to isolate mitochondria. The combined pellets were resuspended in buffer A and centrifuged (25 000×g, 10 min), the lower brown part of the pellet was resuspended in 1 ml buffer C and is referred to as the mitochondria fraction. The supernatant from the 8 000×g centrifugation was further centrifuged (124 000×g, 60 min) and the pellet referred to as internal membranes were suspended in 1 ml buffer C. Each step was performed at 4°C.

### Caveolae enrichment

Plasma membranes (originating from 50–115×10^6^ isolated hepatocytes) prepared as described above were resuspended in 1 ml 0.5 M Na_2_CO_3_ pH 11 with 1 µg/ml pepstatin A, 10 µg/ml leupeptin and 10 µg/ml antipain and sonicated with a probe-type sonifier (soniprep 150) 3×20 sec according to previously well described procedures [15]–[17]. The sonicated plasma membranes were thereafter placed in the bottom of a tube and mixed with 1 ml 90% sucrose in 25 mM MES and 0.15 M NaCl to yield a final concentration of 250 mM Na_2_CO_3_ and 45% sucrose. On top of this solution, 4 ml 35% sucrose in 25 mM MES, 0.15 M NaCl and 250 mM Na_2_CO_3_ pH 11 was added. Finally, 4 ml of 5% sucrose in 25 mM MES, 0.15 M NaCl and 250 mM Na_2_CO_3_ pH 11 was layered on top. The gradient was centrifuged using a SW41 Beckman rotor at 39 000×g for 18–19 hrs at 4°C. From the top of the tube, 1 ml fractions were collected. Before measuring PDE3 activity, pH was neutralized (pH 7–8) using approximately 30 µl of 5.0 M HCl per ml sample. Each step was performed at 4°C.

### Preparation of detergent resistant membranes and Superose-6 chromatography

Isolated mouse hepatocytes (20–100×10^6^) were homogenized in 1 ml buffer A and centrifuged (280×g, 10 min). The supernatant was further centrifuged (175 000×g, 40 min) to obtain a pellet containing total membranes. The pellet was homogenized in 1.5 ml of detergent containing buffer, buffer D (25 mM HEPES pH 7.4, 150 mM NaCl, 1 mM EDTA, 10 mM Na_4_O_7_P_2_, 1% NP-40, 5 mM NaF, 1 mM PMSF, 1 mM Na_3_VO_4_, 1 µg/ml pepstatin A, 5 µg/ml leupeptin and 5 µg/ml antipain). Half of the homogenized pellet was immediately subjected to centrifugation (10 000×g for 10 min, 4°C). The supernatant was filtered (0.2 µm pore size) and thereafter subjected to gel filtration chromatography on a Superose-6 (10/300) column with a separation range of 5–4 000 kDa (Amersham Pharmacia Biotech AB, Uppsala, Sweden) in a fast protein liquid chromatography system. The remaining part of the homogenized pellet from the 175 000×g centrifugation was incubated for 1 h at 4°C and then re-centrifuged (175 000×g for 50 min, 4°C). The subsequent supernatant contained solubilized membranes and the pellet detergent resistant membranes (DRM). The pellet was homogenized in 0.75 ml buffer D and thereafter subjected to centrifugation (10 000×g for 10 min, 4°C). The supernatants containing either solubilized membranes or DRM were filtered (0.2 µm pore size) and thereafter subjected to gel filtration chromatography on a Superose-6 (10/300) column. The column was equilibrated and eluted with buffer D and the flow rate was set to 0.5 ml/min and 0.4 ml fractions were collected. Absorbance at 280 nm was monitored on-line and gel filtration standards (Bio-Rad, Hercules, CA, USA) ranging from 1–670 kDa was used.

### Immuno-electron microscopy

Livers from C57BL/6 mice were perfused in situ with 50 ml 37°C phosphate buffered saline (PBS) followed by 100 ml 1.5% paraformaldehyde, 0.5% glutaraldehyde in 0.1 M phosphate buffer, pH 7.2. Liver pieces were dissected and left in fixative for 1 h. The tissue pieces were washed in 0.1 M phosphate buffer pH 7.2, dehydrated in graded ethanol concentrations to 100% ethanol and embedded in Lowicryl HM120 (TAAB, Reading, UK) as previously described [18], [19]. Ultrathin sections were cut and placed on gold grids. Sections were blocked with PBS (pH 7.2) containing 0.5% bovine serum albumin (BSA) and incubated over night at 4°C with primary antiserum (PDE3B-CT diluted 1∶50 in PBS containing 0.25% BSA and 0.25% Triton X-100) or PBS containing 0.25% BSA and 0.25% Triton X-100 (as negative control). The sections were washed thoroughly in PBS and thereafter incubated for 1 h with gold-conjugated (10 nm diameter gold particles) goat-anti-rabbit IgG (diluted 1∶20, Amersham Pharmacia Biotech AB, Uppsala, Sweden) and washed again in PBS. All sections were contrasted with 0.5% lead citrate and 4% uranyl acetate before examination in a Philips CM10 transmission electron microscope.

### PDE-assay

PDE activity was measured in duplicate in the presence or absence of 3 µM of the specific PDE3 inhibitor OPC3911 [20] (Otsuka Pharmaceuticals Co., Tokyo, Japan), as described previously [10]. The assay was performed at 30°C in a total volume of 300 µl containing 50 mM TES pH 7.4, 250 mM sucrose, 1 mM EDTA, 0.1 mM EGTA, 8.3 mM MgCl_2_, 0.5 µM cAMP, 1 µCi/ml ^3^H-cAMP and 0.6 µg/ml ovalbumin.

### Immunoblot analysis

Different hepatocyte fractions were mixed with Laemmli sample buffer and subjected to SDS-polyacrylamide gel electrophoresis (PAGE) (9–12% acrylamide) before electrotransfer of the proteins to hybond-C extra (Amersham Bioscienses, Buckinghamshire, UK) (fractions from subcellular fractionation) or PVDF membranes. Membranes were blocked for 1 h with 5–8% fat free milk in 20 mM Tris pH 7.6, 137 mM NaCl, and 0.1% Tween-20 before incubation with antibodies as indicated at 4°C over night. The analyses were performed using Super Signal reagents (Pierce, Rockford, USA) and CCD camera (LAS 1 000 Plus, Fuji, Tokyo, Japan) or exposure to Kodak autoradiographic film.

### Preparation of primary hepatocytes

PDE3B KO and WT mice (3–5 months old) were anesthetized with an intraperitoneal injection of midazolam (Dormicum, Hoffman- La Roche, Basel, Switzerland) and a mixture of fluanison/fentanyl (Hypnorm, Janssen, Beerse, Belgium). The livers were subjected to non-recirculating two-step collagenase perfusion. The perfusion was done through the portal vein according to a protocol modified from Carlsson et al [21]. In the first step, Hank's Balanced Salt Solution (HBSS) without calcium and magnesium (Invitrogen Corporation, Carlsbad, CA, USA) supplemented with 2 mM EGTA, 20 mM HEPES and 10 mM NaHCO_3_, pH 7.4, was infused with a pump for 6 minutes with a flow rate of 12–15 ml/min. In the second step, the liver was perfused with William's E medium with Glutamax (Invitrogen Corporation, Carlsbad, CA, USA) supplemented with 50 U/ml, penicillin, 50 µg/ml streptomycin, 0.28 mM sodium ascorbate, 0.1 µM Na_2_SeO_3_ and 0.45 mg/ml collagenase type IV (Sigma) for 6 min at the same flow rate as above. The perfusion solutions were kept in a 37°C water bath and continuously infused with 95% O_2_ and 5% CO_2_. After the perfusion, the livers were excised and the hepatocytes were flushed out from the liver with ice cold William's E medium supplemented as described (without collagenase) and filtered through a 250 µm pore size mesh nylon filter (Sintab Produkt AB, Oxie, Sweden) followed by a 100 µm pore size nylon filter (Falcon, BD Bioscience, Stockholm, Sweden). The cells were washed with William's E medium supplemented as previously described but with the addition of 3 nM insulin (Actrapid, Novo Nordisk, Denmark) and 1 nM dexamethasone. The cells were centrifuged at 50×g for 2 minutes between the washes. The viability of the cells was more than 70% as determined by trypan blue exclusion. Hepatocytes used for triglyceride and RNA extraction were collected at this step and not cultured. For other experiments, hepatocytes were seeded at a density of 10×10^6^ cells/10 cm or 2×10^6^ cells/well in 6-well Primaria™ plates (BD Bioscience, Stockholm, Sweden). The cells were allowed to attach for 4–5 hours, thereafter the medium and the non-attached cells were aspirated and fresh medium added for culture over night.

### Activation of PDE3B in primary hepatocytes

Hepatocytes were cultured overnight on 10 cm Primaria™ dishes and thereafter washed twice with PBS. The cells were treated without or with 10 nM insulin or 10 nM glucagon for 15 minutes in 5 ml DMEM medium without glucose (Invitrogen Corporation, Carlsbad, CA, USA) supplemented with 5 mM glucose, washed in PBS and thereafter scraped and homogenized in homogenization buffer (50 mM TES, 250 mM sucrose, 1 mM EDTA, 0.1 mM EGTA, 1 µg/ml pepstatin A, 10 µg/ml leupeptin and 10 µg/ml antipain, pH 7.4) with a glass/teflon homogenizer. The homogenate was centrifuged (280×g, 10 min) and the supernatant was analyzed for PDE3 activity as previously described. The PDE3 activity was normalized to protein content using Bradford protein assay.

### RNA isolation and quantitative real-time RT-PCR

Non-cultured hepatocytes (10×10^6^cells/ml) (nine month old mice) were homogenized in lysis buffer (RLT buffer, Qiagen, Solna, Sweden) using syringe and needle. The homogenate was either frozen in −80°C until use or total RNA was immediately isolated using RNeasy kit (Qiagen, Solna, Sweden). Primers were designed on the basis of the sequences available at the NCBI gene bank and produced by DNA technologies (Aarhus, Denmark). Primer sequences and accession numbers are presented in Table 1. The real-time RT-PCR analyses were performed on a Miniopticon Thermal Cycler (BioRad, Hercules, CA, USA) and the software MJ Opticon Monitor (BioRad) was used for analysis. SYBR Green I was used as the source of fluorescence and cyclophilin A (CypA) was used as reference gene. The concentration of primers was 400 nM and 250 ng of template was used under the following conditions: 25°C for 5 min, 50°C for 10 min, 95°C for 5 min and then 55 cycles of 94°C for 10 sec, 53°C for 10 sec and 72°C for 1 min. The specificities of the PCR products were confirmed by single dissociation curves. The mRNA expression was calculated using the 2^−ΔΔC(t)^-formula and expressed as arbitrary units in relation to cyclophilinA mRNA expression.

**Table 1:** Primer sequences used for quantitative real-time RT-PCR.

Gene	Primer fwd	Primer rev	Accession nr
Phosphoenolpyruvate carboxy kinase (PEPCK)	tatctggaggaccaggtcaa	tagatctcagcgcatgctgt	NM_011044
Peroxisome proliferator activated receptor gamma (PPARγ)	gaactgcagctcaagctgaa	tgaaggctcatgtctgtctc	NM_011146
Hydroxy-3-methylglutaryl coenzyme A reductase (HMGCR)	gcagtcagtgggaactattgca	cggcttcacaaaccacagtct	NM_008255
Sterol regulatory element binding transcription factor 1 (SREBF-1)	ggagccatggattgcacatt	ggaagtcactgtcttggttgttga	NM_011480

### Glucose production

Hepatocytes were cultured overnight in 6-well plates, washed twice with PBS, and thereafter incubated in Krebs-Ringer bicarbonate buffer (120 mM NaCl, 5 mM NaHCO_3_, 5 mM KCl, 1.2 mM KH_2_PO_4_, 2.5 mM CaCl_2_, 1.2 mM MgSO_4_, 0.2% BSA, 10 mM HEPES, pH 7.2–7.4) supplemented with 5 mM Na-pyruvate and 5 mM Na-lactate as gluconeogenic substrate. The cells were incubated for 4 h after which the culture medium was collected and centrifuged and glucose concentration was determined with a reagent based on glucose oxidase/hydrogen peroxidase enzymatic reaction (DiaSys Diagnostic Systems, Holzheim, Germany). The glucose concentration was normalized to cell density determined as protein content using Bradford protein assay.

### Hepatocyte triglyceride content

Non-cultured hepatocytes (about 2×10^6^ cells) were homogenized in 200 µl William's E medium supplemented with Complete protease inhibitor cocktail (Roche, Bromma, Sweden), 5 mM NaF and 1% Triton X-100. The homogenate (25 or 50 µl) was transferred to a glass tube containing 3 ml chloroform/methanol (2∶1) solution and stored in a N_2_ environment at 4°C overnight. Water (1.5 ml) was added and the tubes were centrifuged at 2000×g for 10 min. After removal of the upper layer the wash step was repeated once. The samples were dried under N_2_ and resuspended in chloroform. Aliquots were transferred into Eppendorf tubes and air-dried. Thesit (Bio Chemika, Sigma-Aldrich, Stockholm, Sweden; 20%, v/w, in chloroform) was added and thereafter the sample was solubilized in water and incubated at 37°C for 10 min. A standard curve was prepared with use of different concentrations of triolein treated in parallel with the samples. Triglyceride reagent (Infinity™, Thermo Electron, Melbourne, Australia) was added according to the manufacturer and absorbance was measured at 510 nm. The triglyceride content was normalized to protein content using Bradford protein assay.

### Cholesterol and protein measurement

Livers previously fixed in formaldehyde (150 mg of dried tissue/sample) were chopped with a scalpel into cubes of around 1 mm^3^. Lipids were extracted with methanol∶chloroform (2∶1) as described in the section “Hepatocyte triglyceride content”. Cholesterol levels were determined by measuring the amount of H_2_O_2_ produced by cholesterol oxidase using Infinity™ Cholesterol Liquid Stable Reagent (Thermo Electron, Melbourne, Australia). This assay was optimized for measuring cholesterol in tissue samples. Protein was measured using a bicinchoninic acid (BCA) protein reagent kit from Pierce (Rockford, USA).

### Statistics

Data are presented as mean±standard error of the mean (SEM) from the indicated number of experiments. Data was analyzed using a two-tailed paired student's t-test (hepatocyte stimulation and glucose output) or a two-tailed non-parametric Mann-Whitney test (triglyceride and cholesterol content and mRNA expression). Differences with a p<0.05 were considered significant. *p<0.05, **<0.01 and ***<0.001.

## Results

### Hepatocyte PDE3B is distributed in plasma membrane and internal membrane fractions

**Figure 1:**
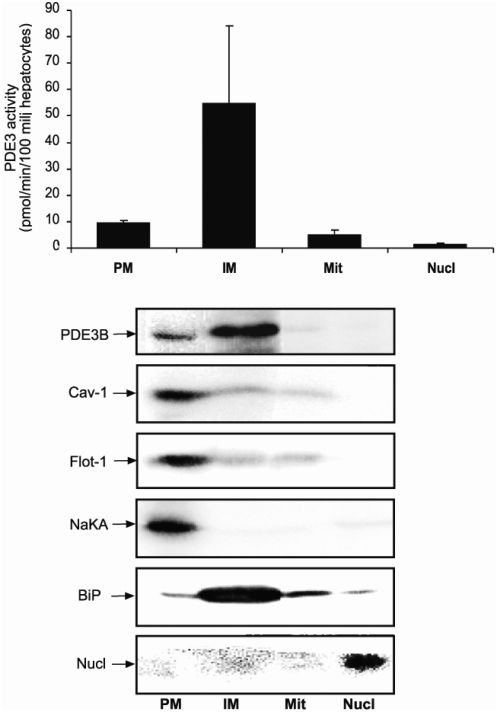
Localization of PDE3B in isolated hepatocytes. Hepatocytes (35–100×10^6^) were isolated from C57BL/6 mice and subjected to subcellular fractionation. Four compartments were isolated; the plasma membranes (PM), internal membranes (IM), mitochondria (Mit) and Nuclei (Nucl). The fractions were subjected to PDE3 activity measurements (a) and immunoblot analysis (b) with antibodies against PDE3B, caveolin-1 (Cav-1, a caveolae marker), flotillin-1 (Flot-1, a lipid raft marker), Na^+^K^+^-ATPase (NaKA, a PM marker), BiP (ER marker) and nucleoporin p62 (Nucl, a nucleus marker). PDE3 activities are shown as mean±SEM and immunoblots as one representative experiment (n = 4). Amount of proteins loaded on the gel: PM, 79 µg; IM, 82 µg; Mitochondria, 31 µg; Nuclei, 31 µg.

To examine the intracellular localization of PDE3B in mouse hepatocytes, we performed subcellular fractionation of isolated mouse hepatocytes, as described in Materials and Methods. Subcellular fractions were first analyzed with regard to subcellular markers; Na^+^K^+^ -ATPase for plasma membrane, BiP (an ER chaperone) for ER and Nucleoporin p62 (nucl) for the nucleus (Figure 1b). As seen in Figures 1a and 1b, PDE3B was found in the plasma membranes and internal membranes, the latter consisting, to a large extent, of ER. According to Figure 1a, the proportion of PDE3B in the plasma membrane and internal membrane fraction is 1∶5. When PDE3B activity in subcellular fractions was expressed per mg protein the following results were obtained for plasma membranes, internal membranes, mitochondria and nucleus, respectively; 5.5±1.7, 10.2±3.2, 4.4±1.2 and 2.9±0.83 pmol PDE3B/mg protein (n = 3, mean±SEM). The subcellular fractions were also analyzed for the caveolae marker caveolin-1 and the lipid-raft marker flotillin-1. Both these proteins were present selectively in the plasma membrane (Figure 1b). PDE4, another major hepatocyte PDE, was mainly found in the cytosolic fraction (data not shown).

### Plasma membrane PDE3B is associated with caveolae in hepatocytes

**Figure 2:**
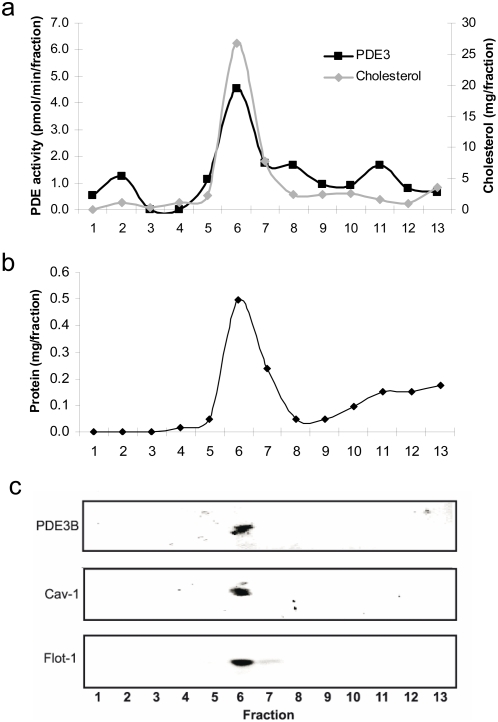
PDE3B is present in caveolae-enriched fractions of hepatocyte plasma membranes. Plasma membranes isolated from mouse hepatocytes (50–120×10^6^) were sonicated in NaCO_3_ and subjected to sucrose gradient fractionation as described in Materials and Methods. Fractions of 0.8 ml were collected starting from the top. The fractions were analyzed for PDE3 activity and cholesterol (a), proteins (b) and subjected to immunoblot analysis (90 µl of each fraction) using antibodies against PDE3B, caveolin-1 (Cav-1) and flotillin-1 (Flot-1) (c). One representative experiment is shown (n = 4).

In order to investigate the possibility that PDE3B is localized to caveolae in hepatocytes, we isolated caveolae from the plasma membrane of isolated mouse hepatocytes. The plasma membranes were sonicated in high concentration of Na_2_CO_3_ and put on a sucrose gradient to separate caveolae and lipid rafts from other components of the plasma membrane fraction. After sucrose gradient fractionation of the sonicated plasma membrane a milky band was detected at the interface of 5 and 35% sucrose. All of the PDE3B activity appeared together with flotillin-1, caveolin-1 and cholesterol in the caveolae/raft enriched fraction (Figures 2a and 2c). This fraction (fraction 6) contained 33% of total proteins whereas factions 7–8 contained 20% and fractions 9–13 contained 42% of total proteins (Figure 2b). When caveolae were enriched from detergent resistant plasma membrane of hepatocytes the same results were obtained (data not shown).

**Figure 3:**
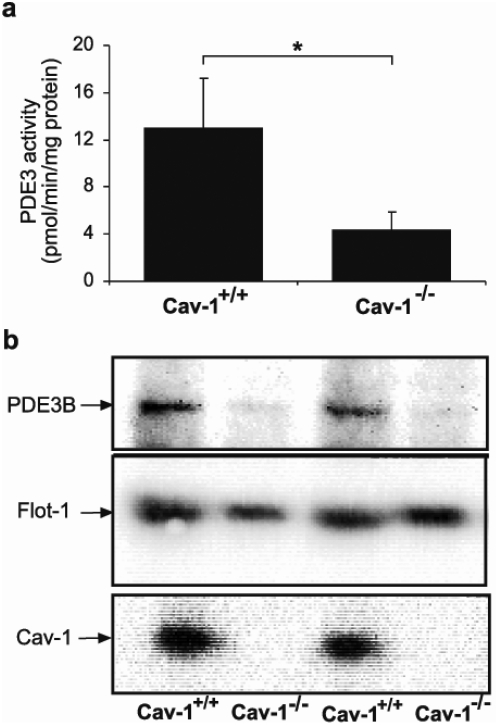
PDE3B protein is reduced in plasma membranes isolated from livers of caveolin-1 KO mice. Plasma membranes isolated from WT and caveolin-1 KO livers were analyzed for PDE3 activity (a) and subjected to immunoblot analysis with antibodies against PDE3B, flotillin-1 (flot-1) and caveolin-1 (cav-1) (b). PDE3 activities (mean±SEM) were significantly different in the two groups (p<0.046). One immunoblot (34 µg protein/lane) with two representative experiments are shown (n = 5).

The importance of caveolin-1 for PDE3B expression was studied using caveolin-1 KO mice. The lack of caveolin-1 results in the absence of caveolae [12]. Thus, we isolated plasma membranes from livers from caveolin-1 KO mice and measured the level of PDE3B protein and activity. As seen in Figure 3, PDE3 protein and activity were significantly lower in plasma membranes from the caveolin-1 KO mice compared to WT mice. In total homogenates from caveolin-1 KO mice PDE3 activity was slightly lower (3.88±0.22) compared to WT mice (4.40±0.65) (n = 3, not significant). These results show that caveolin-1 and caveolae are important for determining the amount of PDE3B protein in the plasma membrane of hepatocytes.

**Figure 4:**
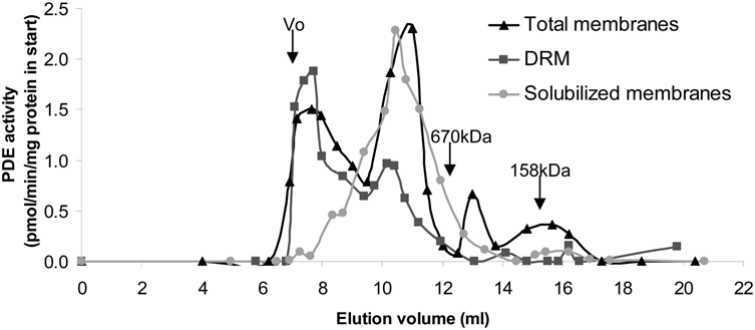
PDE3B is in large size complexes after gelfiltration. Total membranes were prepared from isolated mouse hepatocytes (20–100×10^6^) and treated with detergent. A portion was used to isolate non-solubilized detergent resistant membranes (DRM) and solubilized membranes. The different membranes were subjected to Superose-6 chromatography. Fractions were collected and analyzed for PDE3 activity. One representative experiment is shown (n = 4).

To further study the possible association of PDE3B with caveolae, we took advantage of the fact that caveolae are detergent resistant. Total membranes, solubilized membranes and detergent resistant membranes (DRM) were prepared from mouse hepatocytes. The solubilized membranes and DRM fractions were subjected to superose-6 gel filtration chromatography. PDE3 activity in the total membrane fraction (detergent-treated, 10 000×g supernatant) eluted in two peaks, one with a Mw slightly above 670 kDa (elution volume 10–12 ml) and one corresponding to the void volume (>4 000 kDa, elution volume 7–8 ml) (Figure 4). PDE3 from the solubilized membranes (detergent-treated, 100 000×g supernatant) eluted with the “10–12 ml peak” whereas PDE3 in the DRM (detergent-treated, 100 000×g pellet) eluted primarily with the void volume which may represent PDE3 associated with caveolae and/or lipid rafts. The proportion of total PDE3B activity in the void volume in relation to the “10–12 ml peak” was estimated to be 1∶2.

### PDE3B is associated with smooth endoplasmic reticulum

**Figure 5:**
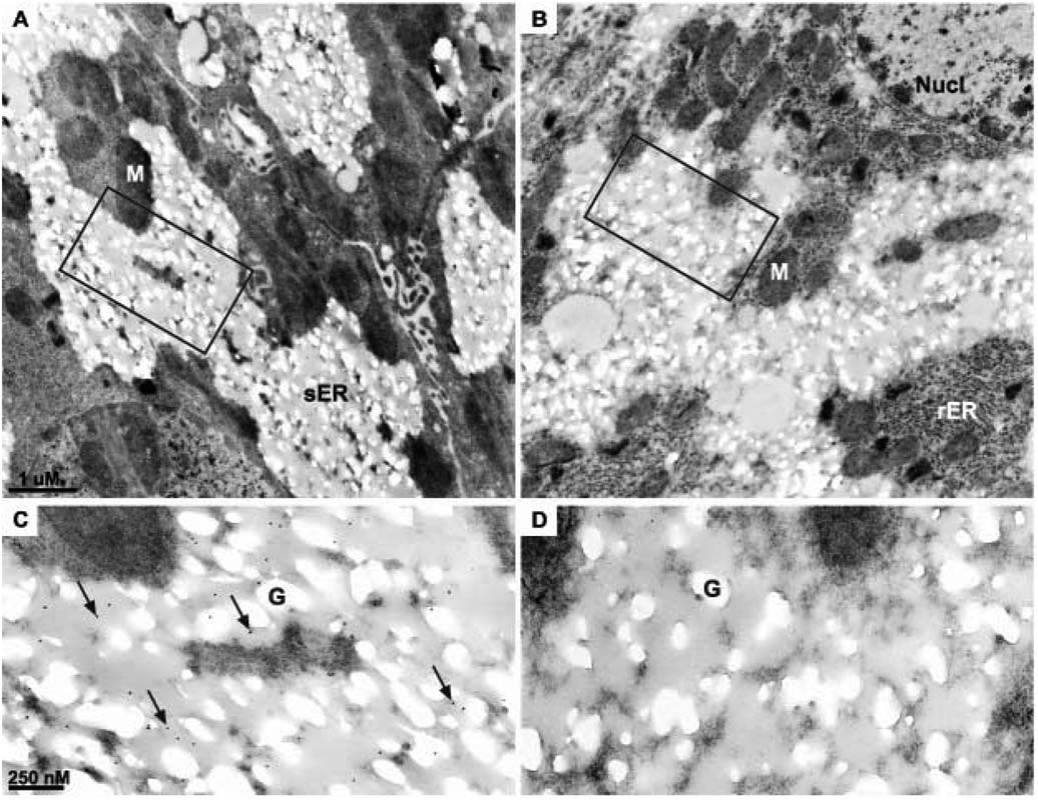
Transmission electron micrographs of mouse hepatocytes; PDE3B is localized to the smooth ER. Sections of lowicryl embedded mouse livers with immunogold labeling for PDE3B (A and C). B and D: negative control with omission of the primary antibody. C and D are higher magnifications of the indicated areas in A and B, respectively. Arrows show examples of gold labeling. Representative micrographs are shown (n = 4). G; Glycogen storage M; Mitochondria, Nucl; nucleus, rER; rough endoplasmic reticulum, sER; smooth ER.

In order to study PDE3B localization in internal membranes at the ultrastructural level, transmission electron microscopy (TEM) with immunogold labeling for PDE3B was employed. Thorough examination of several liver sections from different mouse livers revealed that the majority of the labeling for PDE3B was localized to smooth ER (Figure 5). In addition, weak PDE3B labeling was detected in rough ER as well as in mitochondria (Figure 5). Plasma membranes displayed weak labeling only (data not shown). Control sections displayed randomly scattered labeling only. Smooth ER has previously been shown to be in close association with glycogen particles [22]–[24]. As seen in Figure 5, PDE3B is located in close proximity to the glycogen particles which appear as pale areas among the smooth ER.

### Basal glucose production is elevated in PDE3B KO mice

**Figure 6:**
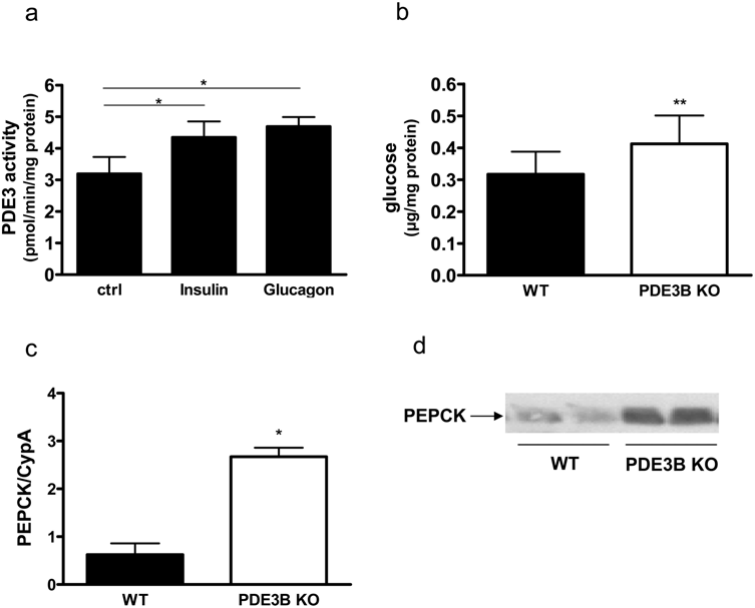
Hormonal regulation and glucose production in mouse hepatocytes. Hepatocytes were isolated from C57BL/6 mice and cultured on Primaria plates. After 18 hours the hepatocytes were incubated for 10 min with 10 nM insulin, 10 nM glucagon or without stimuli (Ctrl). PDE3 activity was measured in total homogenates (a). Values represent mean±SEM (p<0.032 for Insulin, p<0.024 for Glucagon, n = 5). Hepatocytes were isolated from PDE3B KO and WT mice. For glucose production (b) and Western blot analysis of PEPCK (d), the hepatocytes were cultured on Primaria plates over night before the experiment. RNA for PEPCK mRNA expression analysis (c) was isolated from non-cultured hepatocytes. Values are means±SEM. Glucose production: p<0.006, n = 7. PEPCK mRNA: p<0.02, analyzed in duplicate in two independent experiments from two mice of each genotype. PEPCK western blot: one representative experiment is shown, n = 4.

Hepatocytes isolated from C57BL/6 mice were cultured over night and thereafter stimulated without or with insulin or glucagon for 15 minutes. Cell homogenates were analyzed for PDE3 activity. As seen in Figure 6a, stimulation of hepatocytes with either insulin or glucagon induced activation of PDE3 by 35 and 45%, respectively, in agreement with previous results from rat hepatocytes [25]–[26]. Activation of PDE3B by insulin and glucagon has previously been suggested to play a role in hormone-mediated regulation of rat hepatocyte glucose production [27]. Indeed, as shown in Figure 6b, PDE3B KO mouse hepatocytes showed increased glucose production as compared to WT hepatocytes which is in agreement with previously performed clamp studies in these mice [11]. Furthermore, PDE3B KO hepatocytes showed increased mRNA (Figure 6c) and protein levels (Figure 6d) of the key gluconeogenic enzyme phosphoenolpyruvate carboxykinase (PEPCK).

### Triglyceride- and cholesterol contents are increased in hepatocytes isolated from PDE3B KO mice

**Figure 7:**
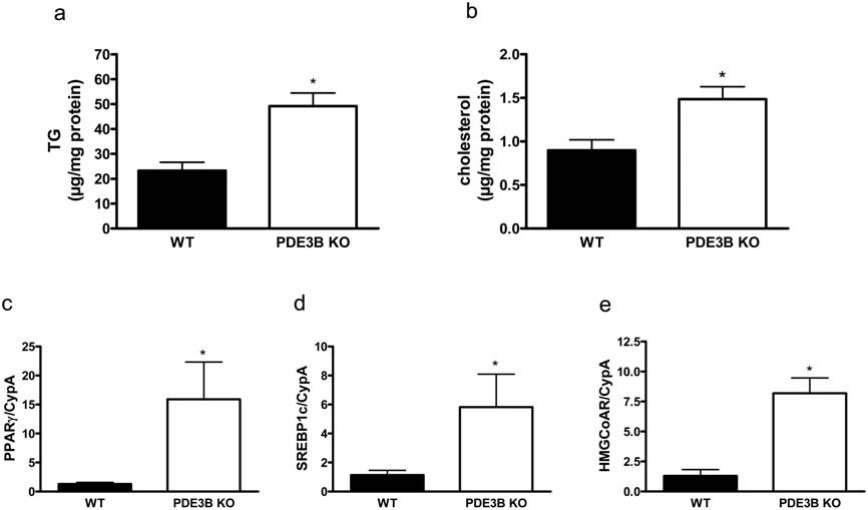
Triglyceride and cholesterol contents are increased in hepatocytes isolated from PDE3B KO mice. Hepatocytes (a, c–e) and liver (b) from PDE3B KO and WT mice were analyzed with regard to triglyceride (a) and cholesterol (b) content. Furthermore, PPARγ mRNA (c), SREBP1c mRNA (d) and HMG CoA reductase mRNA (e) were analyzed in hepatocytes. Values are means±SEM. Triglyceride content was analyzed as duplicate lipid extractions from four animals of each genotype, p<0.03. Cholesterol content was measured in duplicate in six mice of each genotype, p<0.03. mRNA expressions were analyzed in duplicate in two independent experiments from two mice of each genotype.

Triglycerides (Figure 7a) and cholesterol (Figure 7b) were analyzed in isolated hepatocytes. Both triglycerides and cholesterol were increased in PDE3B KO hepatocytes. The increments were associated with elevated levels of peroxisome proliferator-activated receptor γ (PPARγ) and sterol regulatory element-binding protein 1c (SREBP1c) mRNAs, both key lipogenic transcriptional factors (Figures 7c and 7d), as well as mRNA for hydroxyl-3-methylglutaryl coenzyme A (HMG CoA) reductase, a rate limiting enzyme in cholesterol synthesis (Figure 7e).

## Discussion

In this study we demonstrate that PDE3B is localized to caveolae/lipid raft regions in the plasma membrane as well as in smooth ER. Further, we show that PDE3B plays important roles in the regulation of glucose, triglyceride and cholesterol metabolism in hepatocytes.

The localization of PDE3B to caveolae is highly interesting, since caveolae have been suggested to be important in the organization of cellular signalling as well as in lipid synthesis and cholesterol homeostasis [7]. The presence of caveolae in hepatocytes has previously been demonstrated using different approaches. Plasma membranes of isolated rat hepatocytes has been shown to contain sphingolipid-enriched microdomains with high amounts of cholesterol and caveolin-1 [28], and the presence of caveolae in the hepatocyte plasma membrane has been shown directly using rapid-freeze, deep-etching electron microscopy [29]. In agreement with the presence of caveolae in hepatocyte plasma membranes, in this study we detect caveolin-1 and flotillin-1, markers for caveolae and lipid rafts, respectively, specifically in the plasma membrane of isolated mouse hepatocytes and fractionation of sonicated plasma membranes in a sucrose gradient resulted in co-migration of caveolin-1, flotillin-1 and cholesterol. Furthermore, results from caveolin-1 KO mice suggest an important role of caveolin-1 in hepatocyte lipid droplet formation and liver regeneration [30]–[32]. The finding that caveolin-1 KO livers show reduced levels of both PDE3B activity and protein expression indicates a role of caveolin-1/caveolae in the stabilization of the PDE3B protein which has also been demonstrated for the adipocyte PDE3B [5]. Our results demonstrating that PDE3B in hepatocytes is localized to distinct cellular locations, plasma membrane/caveolae as well as smooth ER, is in agreement with recent results in adipocytes [5], [6]. Exactly which is the role for PDE3B at these cellular locations needs to be further investigated.

In this study, we demonstrate increased glucose production and increased level of PEPCK mRNA and protein in hepatocytes from PDE3B KO mice, which is in agreement with previous studies in these mice (clamp studies *in vivo* and PEPCK expression in intact liver) and with studies using PDE3 inhibitors [11], [27]. The increase in glucose production observed in isolated hepatocytes is not extensive and whether this increase can fully explain the increased production of glucose seen *in vivo* in PDE3B KO mice is difficult to know. Although we culture the hepatocytes on Primaria™ dishes to keep as much as possible of the original properties of the cells [33], the hepatocytes most likely have reduced biological responsiveness compared to the *in vivo* situation in PDE3B KO mice. Furthermore, a number of defects related to the storage of triglycerides and cholesterol were identified in hepatocytes from PDE3B KO mice. Thus, we found up-regulation of triglyceride levels in hepatocytes as was the case in PDE3B KO livers [11] and of SREBP1c, one of two transcriptional factors encoded by SREBF1 [34]. The expression of SREBP1c predominates in the liver and has previously been shown to be regulated by insulin and increased intracellular cAMP [34]. In this study we demonstrate a specific role of PDE3B in cAMP mediated regulation of SREBP1c. Nuclear SREBPs is known to interact with cAMP response element binding protein (CREBP)-binding protein (CBP) and PPARγ–regulated coactivator-1β (PGC-1β) which leads to upregulation of fatty acid synthase and suppression of PEPCK gene expression in liver in response to insulin [34]. Cyclic AMP has also been shown to contribute to the regulation of HMG CoA reductase [35], however, little is known regarding involvement of specific PDEs, such as PDE3B, in this process and in the regulation of cholesterol homeostasis. In this study we show that indeed PDE3B seems to be important in this context but the exact signalling pathways in the regulation of HMG CoA reductase and cholesterol synthesis needs to be further evaluated. Thus, in hepatocytes from PDE3B KO mice, increased triglyceride and cholesterol biosynthesis occurred in parallel with elevated gluconeogenesis. At this point we can not completely exclude that decreased breakdown and/or release of triglycerides and cholesterol contribute to the increased levels of the lipids in the PDE3B KO hepatocytes. However, the increased expression of PPARγ, SREBP1c and HMG CoA reductase together with previous results [11] showing increased expression of fatty acid synthase (FAS) in PDE3B KO liver, indicate that increased biosynthesis contribute to the fatty liver phenotype. Indeed increased accumulation of fat in the liver is highly relevant in the context of type 2 diabetes (T2D) and the metabolic syndrome [36], [37]. It has been estimated that 70–80% of T2D patients have non alcoholic fatty liver disease. This disease covers a spectrum of liver diseases from steatosis to nonalcoholic steatohepatitis and cirrhosis. Indeed, increased fat accumulation in the liver appears to be a marker of hepatic insulin resistance and a close correlate of all components of the metabolic syndrome independent of obesity. The role of defect/dysregulated PDE3B in fatty liver development needs to be further investigated.

In summary, we demonstrate that, in mouse hepatocytes, PDE3B is localized in caveolae/raft regions in the plasma membrane as well as in smooth ER and that the enzyme has an important role in the regulation of triglyceride, cholesterol and glucose metabolism in these cells.
